# Honokiol in glioblastoma recurrence: a case report

**DOI:** 10.3389/fneur.2023.1172860

**Published:** 2023-06-22

**Authors:** Ce Wang, Zehao Cai, Yue Huang, Xinrui Liu, Xing Liu, Feng Chen, Wenbin Li

**Affiliations:** ^1^Department of Neuro-Oncology, Cancer Center, Beijing Tiantan Hospital, Capital Medical University, Beijing, China; ^2^Human Brain and Tissue Bank, China National Clinical Research Center for Neurological Diseases, Beijing Tiantan Hospital, Capital Medical University, Beijing, China; ^3^Department of Neuropathology, Beijing Neurosurgical Institute, Beijing, China

**Keywords:** glioblastoma, liposomal honokiol, blood–brain barrier permeability, clinical trial, tumor progression

## Abstract

Glioblastoma is the most common and aggressive primary tumor in the central nervous system. There is no standard of care for patients with recurrent GBM. Honokiol is a pleiotropic lignan and has the potential to be a potent and safe anticancer agent in human GBM when it is encapsulated by liposomes. We report an efficient and safe response to three phases of treatment with liposomal honokiol in a patient with recurrent glioblastoma.

## 1. Introduction

Glioblastoma (GBM) is the most common and aggressive primary tumor in the central nervous system (1). Surgery followed by radiotherapy (RT) with concurrent temozolomide (TMZ) and adjuvant TMZ with or without tumor-treating fields (TTF) is the standard of care for patients with newly diagnosed GBM (2). These patients' median overall survival time is 20.9 months when TTF is administrated (3). However, there is no standard of care for patients with recurrent GBM for whom a clinical trial has been added to the National Comprehensive Cancer Network (NCCN) guidelines as a preferred regimen (4). The development of new therapies requires that clinical trials accrue efficiently and yield relative results (5). Unfortunately, a majority of early clinical trials of chemotherapies and molecularly targeted treatments in patients with GBM constituted defeats due to the inadequate blood–brain barrier (BBB) permeability of agents (6). The increasing focus on permeability has resulted in some notable clinical successes in recent years.

Honokiol is a pleiotropic lignan that can be isolated from the plant *Magnolia grandiflora* (7). It has extensive therapeutic actions such as antimicrobial, neuroprotective, antispasmodic, anti-tumorigenic, anxiolytic, and other actions (8). There are several antitumor actions of honokiol against glioma cells, such as inhibition of cell viability and migration, induction of apoptosis, and suppression of ERK and AKT signaling cascades. It also lowers EGFR and CD133 (9). Furthermore, honokiol can elevate DNA fragmentation and enhance apoptosis of cells induced by TMZ, which is used in the standard care of patients with primary GBM (10). Honokiol can inhibit the growth of GBM by upregulating M1 macrophages and limiting M2 phenotypic macrophages (11). Some investigations of the potential toxic properties of honokiol employed only the concentrated magnolia bark extract (MBE) instead of purified compounds. *In vitro* and *in vivo* studies showed that concentrated MBE has no mutagenic and genotoxic potential (12). Honokiol was employed in conjugation with glucuronic acid in order to increase hydrophilic properties and facilitate excretion. A half-life period of honokiol is ~40–60 min in plasma when injected in rats at 5–10 mg/kg (i.v.) (13, 14). Honokiol has the potential to be a potent and safe anticancer agent in human GBM.

Although honokiol can potentially be an anticancer agent, its extreme water insolubility inhibits its delivery to glioma at an effective concentration (15). It is feasible to use liposomes to encapsulate the honokiol to make it soluble and alter blood–brain barrier permeability. This report aims to assess the safety, tolerability, and efficacy of liposomal honokiol (Lip-HNK) in a patient with GBM, as determined by clinical observation. Using liposomes to encapsulate the honokiol is feasible to make it soluble.

## 2. Case report

A 36-year-old man was admitted to the local hospital on 8 March 2020, with a history of limb twitch that lasted for 10 min. He was investigated with magnetic resonance imaging (MRI), showing a mass in the left frontal lobe. After the resection of a left frontotemporal craniotomy tumor in September 2020, the postoperative MRI showed a residual disease. The histopathological examination was compatible with the diagnosis of GBM. Molecular testing was a wild-type IDH1 R132, a wild-type IDH2 R172, and a mutated TERT C228T. Based on these results, from October 2020 to November 2020, the patient was given postoperative RT for a total dose of 60 Gy, combined with TMZ at the dose of 100 mg/day, according to the STUPP regimen. Two cycles were administered. On 6 January 2021, a follow-up craniocerebral MRI showed the existence of abnormal enhancement shadows in the left frontal lobe, suggesting tumor recurrence.

**Figure 1:**
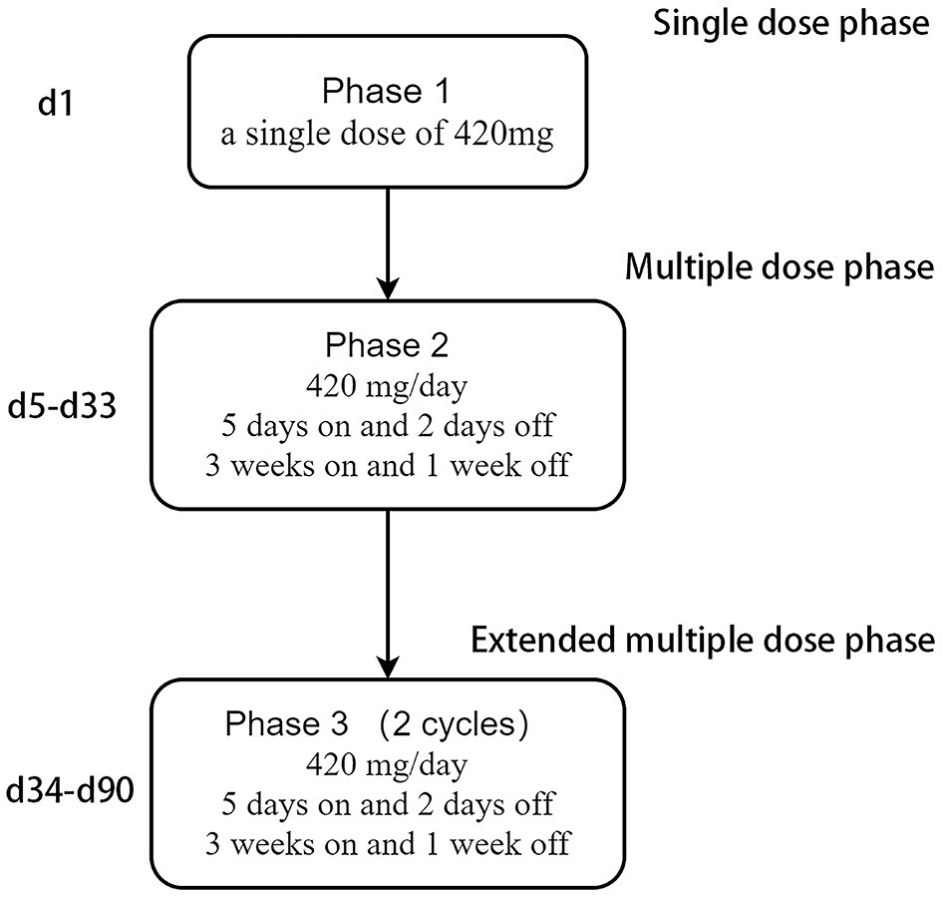
Therapy schedule of liposomal honokiol.

**Figure 2:**
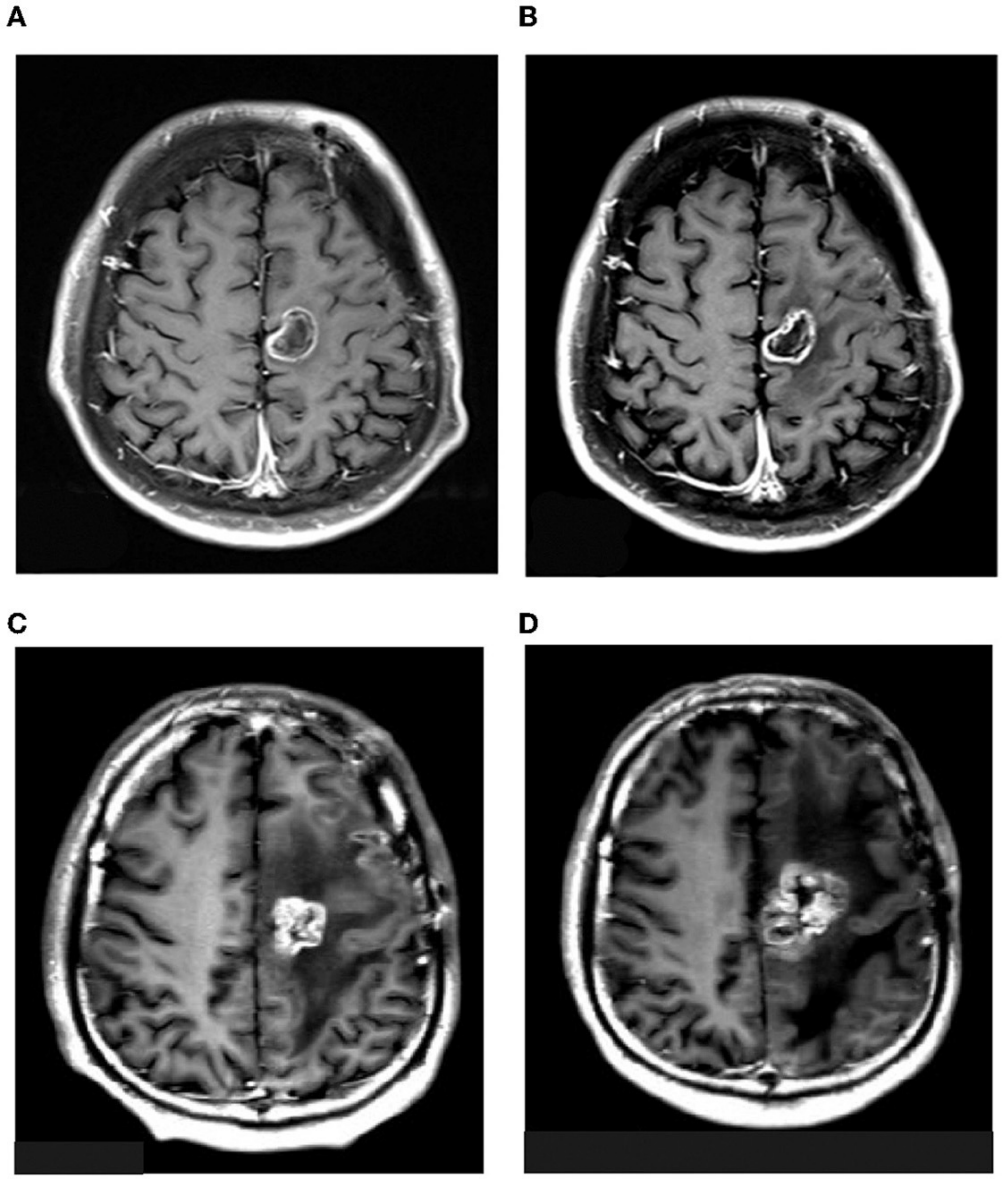
MRI images of the patient on 3rd March **(A)**, 21st May **(B)**, 4th August **(C)**, and 15th November **(D)** 2021.

On 3 March 2021, liposomal honokiol (Lip-HNK) was given to this patient with a single dose of 420 mg (i.v. 250 ml for 2 h). Clinical symptoms were assessed after 3 days, and no side effect was reported. Then, the patient received the next phase of therapy. Every 4 weeks was defined as one cycle of subsequent therapy. Treatment with Lip-HNK was started at 420 mg/day (5 days on and 2 days off) in the first 3 weeks. Then, the treatment was paused to assess the drug safety and the patient's condition after the 4th week. Considering Lip-HNK was effective in the management of this patient with GBM, physicians prolonged the therapeutic regimen. Lip-HNK was given for additional two cycles (the same dose as before). The therapy schedule is shown in Figure 1. A new brain MRI after more than 2 months of therapy, on 21 May 2021, demonstrated no significant change in the lesion size in the left frontal lobe (Figure 2). The patient was sorted into the stable disease (SD) category in accordance with the RANO criteria. Lip-HNK was tolerated well during treatment with no serious adverse effects, and the Karnofsky Performance Status (KPS) was always ≥70 points. There were no abnormalities in ALT, AST, and Cr before and after each treatment, indicating no hepatorenal toxicity of Lip-HNK. The patient's WBC, RBC, and PLT were also in the normal range. We did not observe apparent hematological toxicity.

On 4 August 2021, a follow-up craniocerebral MRI showed a mixed signal shadow and a pronounced surrounding edema in the left frontal lobe, while a T1-CE image showed a ring enhancement (Figure 2C). We administered chemotherapy with cis-platinum combined with temozolomide. Two cycles were administered as MRI showed the existence of tumor progression. On 15 November, the patient was given bevacizumab combined with etoposide and carboplatin because of another tumor progression (Figure 2D). After 19 months of the initial diagnosis, he ultimately passed away from significant clinical deterioration.

**Figure 3:**
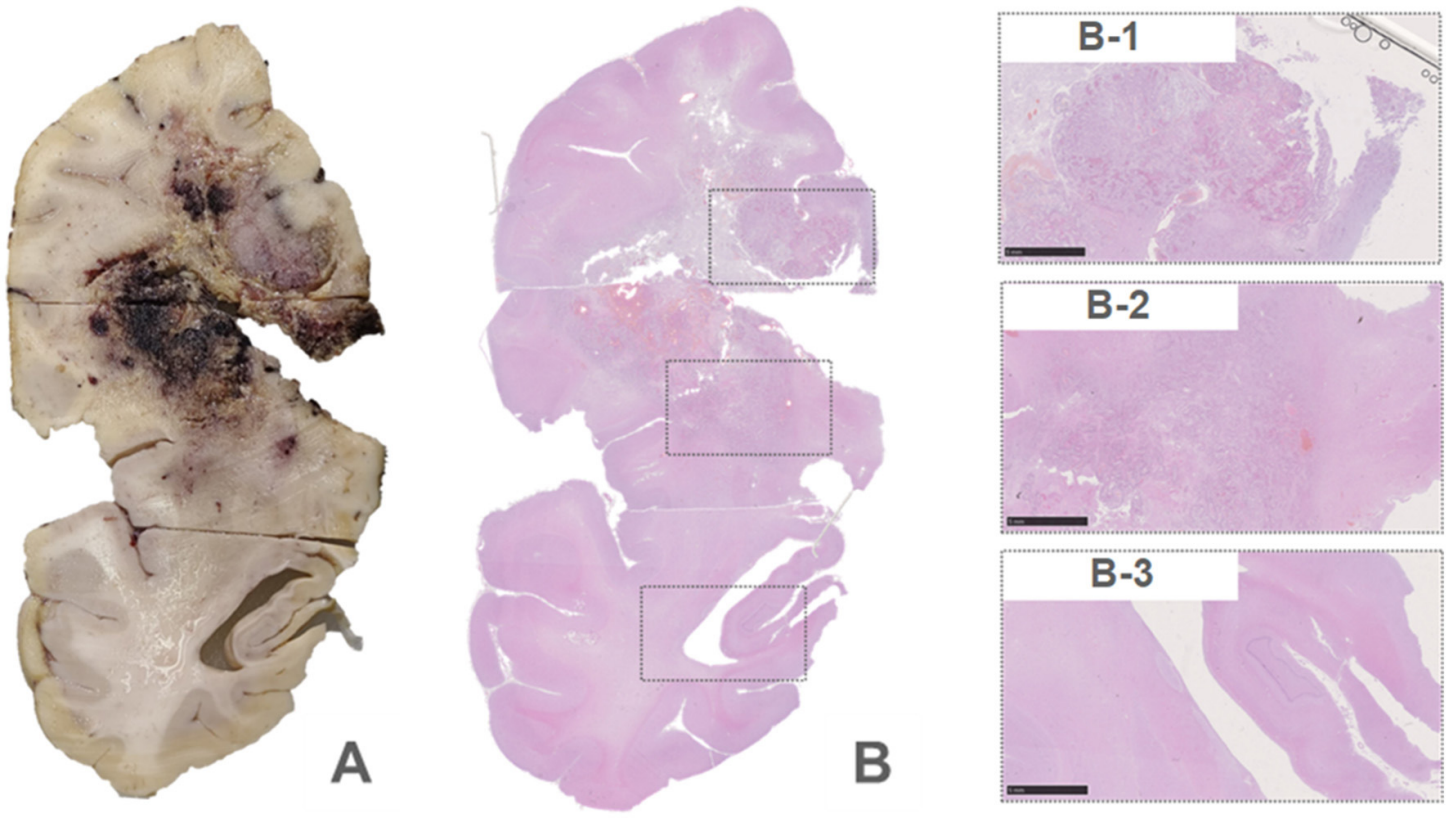
Brain biopsy of the patient **(A)**. The H&E staining of the same site **(B)**.

**Figure 4:**
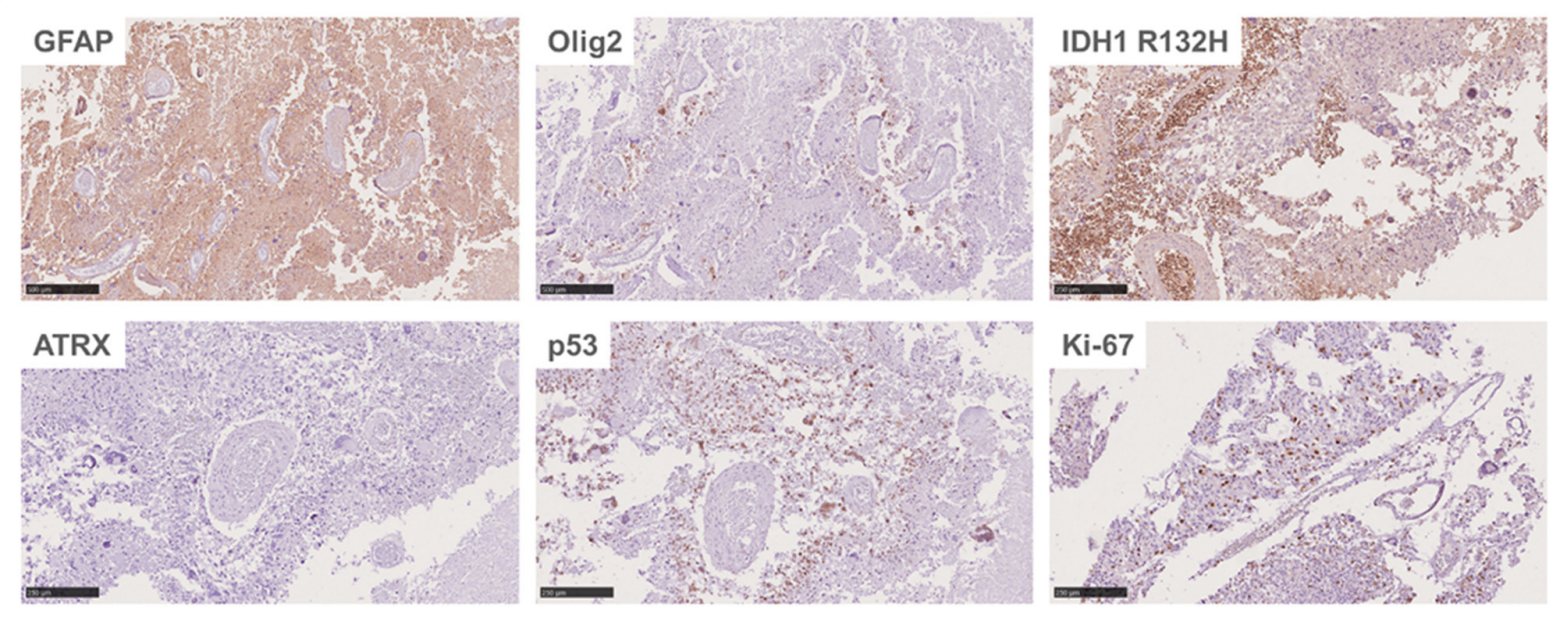
Immunohistochemical stains of the patient's brain.

An autopsy was performed after death. Lesions were found in the frontal lobe, temporal lobe, and bilateral callosum (Figure 3A). A large lesion (2.3 × 1.5 cm) was identified, affecting the cortex of the frontotemporal lobe. The ventricle was surrounded by necrotic and hemorrhagic tissue. On H&E staining, all lesions demonstrated the classic appearance of GBM, including nuclear atypia, palisading necrosis, and microvascular proliferation (Figure 3B). The tumor spread from the white matter to the cortex, where it formed nodules. Tumor invasion was also observed in the subarachnoid space. Immunohistochemical stains were positive for GFAP, Olig-2, and P53 and negative for IDH1 R132H, and ATRX. Moreover, a high Ki-67 expression (30%) was found in the tumor region (Figure 4).

## 3. Discussion

To the best of our knowledge, this case study is the first report of the adjunctive use of honokiol in a patient with GBM, providing evidence of its possible beneficial effects. The patient in this case study was given honokiol at the first disease progression and obtained an overall survival of up to 15 months, which is considerably longer than 9.3 months in patients with recurrent GBM given bevacizumab (16). Notably, there were no severe side effects that led to treatment discontinuation. In particular, Lip-HNK did not cause hematologic toxicities such as neutropenia, anemia, or thrombocytopenia, which are common in chemotherapy-induced myelosuppression. Beyond a stable disease maintained during Lip-HNK treatment, it seemed that Lip-HNK did not markedly affect the efficacy of subsequent chemotherapies. Eventually, the patient died due to tumor dissemination. Autopsy studies described that ~25% of patients with GBM have evidence of spinal subarachnoid seeding, while the prognosis of GBM patients with dissemination is bleak and usually leads to fatal outcomes (17–19).

Our study has been innovative in some ways. We tried to give patients with the GBM plant extract for treatment, which has multiple antitumor effects, instead of traditional chemotherapeutic and targeted agents. Lip-HNK with higher BBB permeability was demonstrated, while the blood–brain barrier has contributed to the lack of progress in GBM treatment over the past 15 years. The therapeutic regimen was divided into several stages to ensure the therapeutic effect for the patient.

Being a potent anticancer agent in various human tumors, honokiol remarkably inhibits tumor growth (20). Lip-HNK can induce lysosomal degradation of Hsp90 client proteins in gefitinib-resistant NSCLC cells (21). It also provides an effective approach to inhibit tumor growth in cisplatin-resistant human ovarian cancer (15). Lip-HNK induces ROS-mediated apoptosis and autophagic inhibition in medulloblastoma (22). Lip-HNK enhances the apoptosis of breast cancer cells induced by Adriamycin and inhibits the proliferation of breast cancer cells both in *vitro* and *in vivo* (23). Currently, Lip-HNK has entered phase I clinical trials for the treatment of GBM and other tumors.

## 4. Conclusion

In conclusion, honokiol might be a new potential treatment option for patients with recurrent GBM. More studies should be performed in an adequately powered population to evaluate the efficacy and safety.

## Data availability statement

The original contributions presented in the study are included in the article/supplementary material, further inquiries can be directed to the corresponding author.

## Ethics statement

The studies involving human participants were reviewed and approved by the Medical Ethics Committee of Beijing Tiantan Hospital. The patients/participants provided their written informed consent to participate in this study. Written informed consent was obtained from the individual(s) for the publication of any potentially identifiable images or data included in this article.

## Author contributions

CW and ZC were the major contributors to writing the manuscript. XinruiL and XingL contributed to the image analysis. YH contributed to the immunohistochemical analysis. FC and WL contributed to checking the manuscript. All authors read and approved the final manuscript.
